# Postprandial glycemic control during gestational diabetes pregnancy predicts the risk of recurrence

**DOI:** 10.1038/s41598-018-24314-1

**Published:** 2018-04-20

**Authors:** Naama Schwartz, Manfred S. Green, Enav Yefet, Zohar Nachum

**Affiliations:** 1 School of Public Health, University of Haifa, Haifa, Israel; 2 Epidemiology & Statistics, Emek Medical Center, Afula, Israel; 3 Department of Obstetrics and Gynecology, Emek Medical Center, Afula, Israel; 4 The Bruce Rappaport Faculty of Medicine, Technion - Israel Institute of Technology, Haifa, Israel

## Abstract

In this study we aimed to explore the significance of glycemic control during gestational diabetes mellitus (GDM) pregnancy in predicting recurrence as this is unknown. A retrospective population-based cohort study of women with first diagnosed GDM pregnancy was conducted. A total of 426 women with 4,226 glucose charts were obtained. Daily glucose values were collected from the glucose charts. Non-parametric (LOWESS) regression was used to present the glucose measurements along the gestational weeks. The analyses revealed that the 2-hour postprandial levels among women with GDM recurrence were substantially higher throughout gestation (PR = 1.89 [95% CI: 1.33, 2.73] for every 20 mg/dl increase). In a multivariable log-binomial regression, the mean postprandial glucose was significantly associated with GDM recurrence (*p* = 0.017) after adjusting for maternal age, family history of diabetes, insulin use, and inter-pregnancy interval (PR = 1.04 [95% CI: 1.01, 1.07]). The study conclusion is that tighter postprandial glycemic control should be considered. Future studies should explore tighter cutoffs of the 2-hour postprandial glucose.

## Introduction

Women diagnosed with gestational diabetes mellitus (GDM) are at increased risk for a variety of adverse birth outcomes^1–3^. In addition, GDM is also a significant predictor for type 2 diabetes^4^. After pregnancy, 5% to 10% of women with GDM are found to have type 2 diabetes, and women with GDM have a 20% to 50% probability of developing diabetes within 5 to 10 years following GDM pregnancy^5^.

About 50% of the women with GDM will have recurrent GDM at their consecutive pregnancy^6^. Reported risk factors for GDM recurrence include maternal age, ethnicity, BMI, weight gain between pregnancies, insulin use, parity, macrosomia, inter-pregnancy interval (IPI), and the oral glucose tolerance test (OGTT) levels^7–9^. Several studies have examined the relevance of OGTT levels as risk factors^9–17^ where the mean values of the OGTT results among women with and without GDM recurrence were compared; except for the 3-hour post glucose load results, the OGTT results were consistently significant. Nonetheless, no study has examined the accuracy of the OGTT levels in predicting GDM recurrence, or presented cutoff values for an increased risk for GDM recurrence.

While the glucose challenge test (GCT) and the OGTT (usually done at 24–28 gestational weeks) represent baseline characteristics of the women before glycemic control was initiated, they contribute less information than the glucose levels in the later third trimester. During the second and third trimesters it is customary to monitor glycemic control using daily glucose charts comprising blood glucose measurements before and after meals. Hypothetically, poor glycemic control as manifested by the daily glucose charts might be associated with GDM recurrence in the future. An initial examination of this hypothesis was explored in a relatively small and old (1998) study, where patients were asked to record at least 4 measurements of glucose per day in a “daily glucose diary” during the second and third trimesters. It was found that among other risk factors, the third trimester mean glucose levels were higher in women in whom GDM recurred in the next pregnancy compared with women without GDM recurrence^17^. No accuracy analysis was presented and no other study further explored this hypothesis. With regard to HbA_1c_, Nohira *et al*^14^. and Schwartz *et al*^8^. did find it to be significant risk factor for GDM recurrence. To our knowledge, no study presented the glycemic control accuracy in predicting GDM recurrence.

Therefore, the main objective of the current study was to explore the association and accuracy of the glucose levels during the third trimester in predicting GDM recurrence. In our secondary objectives, the OGTT significance and accuracy were also examined.

## Materials and Methods

A retrospective cohort study of women with GDM who delivered at Emek Medical Center was conducted. The study population consisted of women with first GDM diagnosis (i.e., index pregnancy) who delivered at Emek Medical Center between 1991 and 2012 and had at least one consecutive birth at the same medical center. We included women with well-documented GDM (according to GCT/OGTT) in both relevant pregnancies, who were monitored and reported their glucose levels during the index pregnancy. Women with preexisting diabetes or women who developed diabetes between pregnancies were excluded.

Emek Medical Center serves a population of about 500,000 people from the cities, towns, and villages in the north east of Israel. All pregnant women in Israel are screened for GDM by undertaking GCT where the women’s plasma glucose is tested after a 50 g oral glucose load between 24 and 28 weeks of gestation. Women are referred for an OGTT if the plasma glucose concentration one hour later is ≥ 140 mg/dl. GDM is diagnosed when two or more abnormal values are presented on a 3-hour 100 g glucose tolerance test using the Carpenter and Coustan criteria^18^ [0 h 95, 1 h 180, 2 h 155, 3 h 140 mg/dl], or one abnormal value using the 1979 National Diabetes Data Group (NDDG)^19^ [0 h 105, 1 h 190, 2 h 165, 3 h 145 mg/dl]. Since the difference between the two criteria is only in the OGTT interpretation, both criteria were used simultaneously and GDM diagnosis was established if at least one of them was fulfilled;^18,19^ GDM is also diagnosed with a GCT value of 200 mg/dl or higher. Once the patient was diagnosed with GDM, she continued with close pregnancy monitoring and glycemic control at the Diabetes in Pregnancy Clinic in the Fetal Maternal Unit of Emek Medical Center. Tight glycemic control was maintained by using dietary and medical intervention as needed. The criteria that were used to guide insulin therapy treatment were preprandial glucose ≥ 95 mg% or 2-hour postprandial glucose value ≥ 120 mg%. The basis of the diagnosis and routine treatment was not changed throughout the study period and all the patients received equal medical service regardless of their level of medical insurance. All the information was obtained from the women’s medical records, laboratory systems, gestational diabetes clinic files, and delivery records.

**Figure 1:**
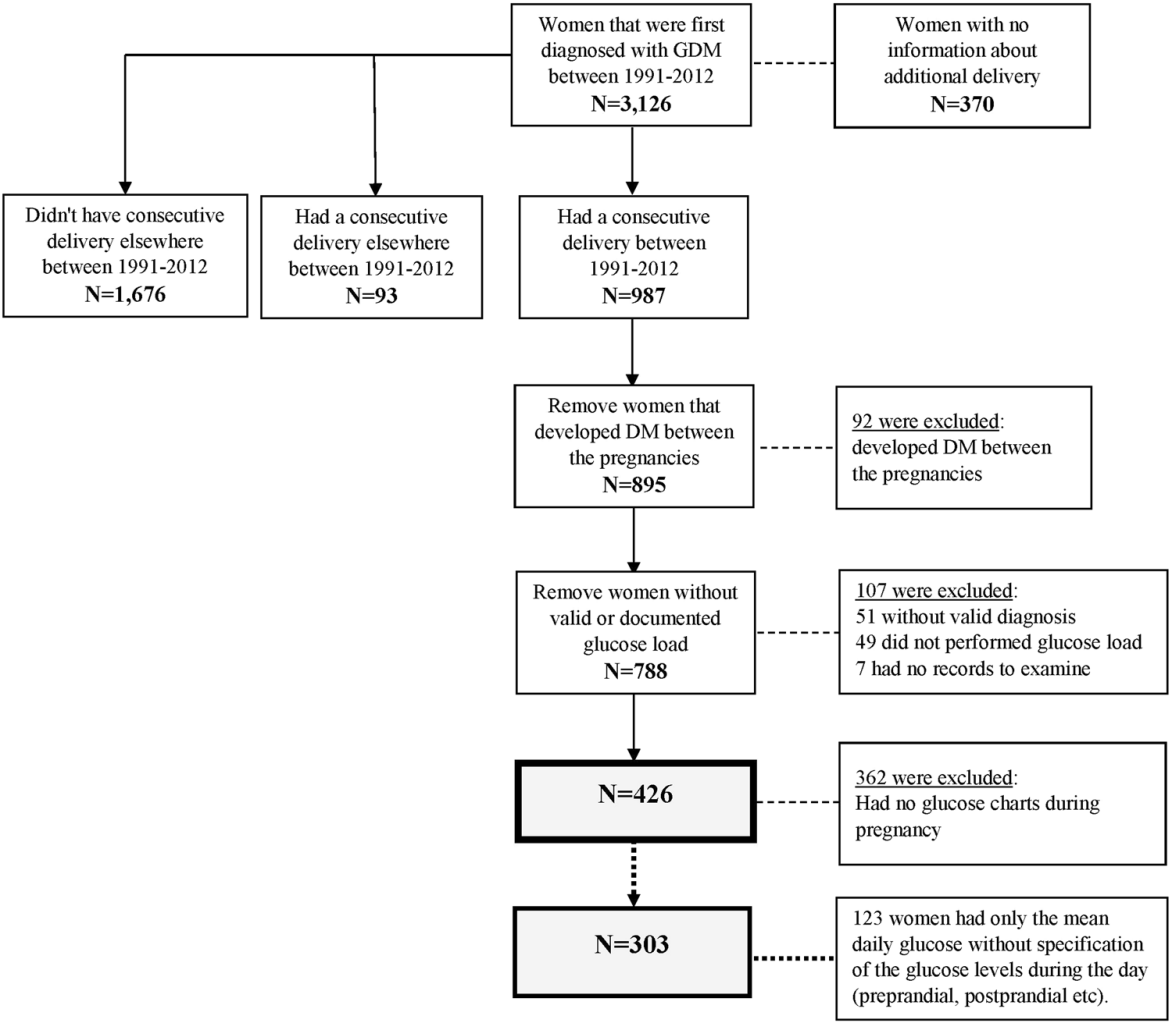
The study flow chart.

The women were identified by utilizing the hospital records using the International Classification of Diseases, Ninth Revision, Clinical Modification (ICD9-CM) code 648.8 (“abnormal glucose tolerance of mother complicating pregnancy childbirth or the puerperium”). Women with 250 and/or 648.0 codes (“Diabetes mellitus” and “Diabetes mellitus complicating pregnancy childbirth or the pueperium”, respectively) at the study period were excluded (Fig. 1). Of the identified 895 women, a total of 107 were excluded. Specifically, we excluded 51 due to invalid GDM diagnosis (misdiagnosis based on the GDM criteria), 49 did not have a documented OGTT, and 7 had no physical records at all. Of the 788 women, 426 had information on the mean glucose levels and of them, 303 had specific preprandial and postprandial glucose information.

### Demographic and clinical data

According to the women’s medical history and the delivery outcome, the following information was obtained at the index pregnancy: maternal age, ethnicity (Jewish or Arab), number of pregnancy, family history of diabetes, GDM diagnosis week, OGTT levels, and neonatal birth weight.

### Laboratory and Glucose monitoring

After completing a full medical history questionnaireing, the women received a glucose meter and were instructed to test their glucose levels 7 times daily: after an overnight fast, preprandial (2 measurements), postprandial (3 measurements), and at bedtime (E-Table 1). Patient follow up including inspection of the daily glucose charts results (visits/telephone/fax) was scheduled up to every week. Complete blood count, HbA_1c_, fructosamine, and chemistry were obtained every month.

**Table 1:** Baseline characteristics of the 426 women according to GDM recurrence.

	Total N = 426	No GDM recurrence N = 169	GDM recurrence N = 257	*p*-value	PR [95% CI]
Maternal age (years)	29.8 ± 4.8	29.1 ± 4.8	30.3 ± 4.8	0.007	1.11 [1.03, 1.19]^a^
	[29.5]	[28.5]	[30.3]		
Maternal age					
<30 years	228	106 (46%)	122 (54%)	0.002	1
≥30 years	198	63 (32%)	135 (68%)		1.27 [1.09, 1.49]
Pre-pregnancy BMI (kg/m²)	26.8 ± 4.8	25.8 ± 4.5	27.3 ± 4.9	0.0005	1.02 [1.01, 1.04]
	[26.3]	[25.1]	[27]		
Parity	2.4 ± 1.8	2 ± 1.6	2.6 ± 1.9	<0.0001	1.04 [1.03, 1.06]
	[2]	[1]	[2]		
Multiparous					
No	194	94 (48%)	100 (52%)	0.001	1
Yes	232	75 (32%)	157 (68%)		1.31 [1.12, 1.54]
Family history of diabetes mellitus					
No	171	83 (49%)	88 (51%)	0.004	1
Yes	255	86 (34%)	169 (66%)		1.29 [1.09, 1.53]
GDM diagnosis week	28.8 ± 4.9	29.3 ± 4.6	28.4 ± 5	0.047	0.98 [0.97, 0.99]
	[28.4]	[28.9]	[28.0]		
OGTT: Fasting (mg/dl)	91 ± 14	89 ± 13	92 ± 15	0.03	1.12 [1.01, 1.24]^b^
	[89]	[88]	[90]		
OGTT: 1-h post glucose load (mg/dl)	202 ± 25	197 ± 25	205 ± 24	0.002	1.09 [1.03, 1.15]^b^
	[200]	[195]	[202]		
OGTT: 2-h post glucose load (mg/dl)	161 ± 33	155 ± 30	164 ± 34	<0.0001	1.07 [1.05, 1.08]^b^
	[162]	[159]	[166]		
OGTT: 3-h post glucose load (mg/dl)	104 ± 39	104 ± 41	105 ± 38	0.86	1.00 [0.96, 1.04]^b^
	[99]	[99]	[98]		
HbA_1c_	5.4 ± 0.6	5.4 ± 0.7	5.4 ± 0.6	0.35	1.05 [0.95, 1.17]
	[5.3]	[5.3]	[5.4]		
Fructosamine	180 ± 18	176 ± 17	182 ± 18	0.003	1.12 [1.04, 1.12]^b^
	[179]	[176]	[181]		
Insulin use					
No	242	118 (49%)	124 (51%)	<0.0001	1
Yes	184	51 (28%)	133 (72%)		1.41 [1.21, 1.64]
Mean overnight fasting glucose levels during the index pregnancy^c,d^	85.6 ± 10.4 [85.6]	85.6 ± 10.1	85.7 ± 10.5	0.8993	1.01 [0.85, 1.20]^b^
		[85.6]	[85.5]		
Mean preprandial glucose levels during the index pregnancy^c,d^	87 ± 11	87 ± 9.8	86 ± 11	0.2487	0.78 [0.5, 1.23]^b^
	[86]	[88]	[86]		
Mean postprandial glucose levels during the index pregnancy^c,d^	109 ± 15	105 ± 13	111 ± 15	0.0005	1.89 [1.33, 2.73]^b^
	[107]	[103]	[110]		
Mean glucose levels during the index pregnancy^c^	95 ± 10	94 ± 10	96 ± 10	0.08	1.11 [0.99, 1.25]^b^
	[94]	[92]	[95]		
Birthweight (gr)	3316 ± 513	3313 ± 482	3318 ± 532	0.93	1.004 [0.93, 1.09]^e^
	[3346]	[3338]	[3354]		
BMI gain between	1.2 ± 2.3	0.9 ± 2.4	1.5 ± 2.2	0.01	1.04 [1.01, 1.07]
the pregnancies (Kg/m²)	[1.2]	[0.5]	[1.6]		
IPI (months)	35.9 ± 20.9	31.4 ± 16.8	38.9 ± 22.7	<0.0001	1.12 [1.10, 1.14]
	[31]	[28]	[35]		
IPI ≤24					
months	147	67 (46%)	80 (54%)	0.08	1
>24 months	279	102 (37%)	177 (63%)		1.17 [0.98, 1.39]

Continuous variables are presented with mean  ±  standard deviation [median].

PR = prevalence ratio; CI = confidence interval; IPI = inter-pregnancy interval; GDM = gestational diabetes mellitus; OGTT = oral glucose tolerance test; BMI = body mass index; HbA_1c_ = Hemoglobin A1c.

^a^For every 5 year increase.

^b^For every 20 units increase.

^c^Adjusted for the number of glucose days reported.

^d^Included only 303 women with specified glucose levels.

^e^For 500 gr increase.

### Information from between the pregnancies

BMI gain between the pregnancies was calculated by subtracting the pre-pregnancy BMI of the index pregnancy from the pre-pregnancy BMI of the consecutive pregnancy. The IPI was calculated according to the time interval (months) between deliveries.

The outcome variables were obtained by exploring the GCT and OGTT at the consecutive pregnancy and determining whether the woman had GDM recurrence or not.

### Study variables

The primary outcome was GDM recurrence (yes/no), which was determined according to the GDM diagnosis criteria in the index pregnancy and the subsequent pregnancy. The exploratory variables at the index pregnancy were: maternal age, pre-pregnancy BMI, parity, family history of diabetes, gestational age at GDM diagnosis, OGTT levels (fasting, 1, 2, and 3 hours post 100 gr glucose load), HbA_1c_, fructosamine, mean glucose levels (daily, preprandial, postprandial), neonatal birth weight, BMI gain, and IPI measured between the index pregnancy and the subsequent pregnancy.

### Power and Statistical Analysis

The area under the receiver operating characteristic (ROC) curve (AUC) is a popular summary measure of the accuracy of a test/marker. When the AUC is 50% or less, it is concluded that the result is not significantly different from random guessing, which is represented by the diagonal line in the preceding ROC plot^20^. We wanted to test if the glucose level had some accuracy (AUC ~60%). We determined minimum power of 80% with alpha 5%. Also, we assumed that the percent of GDM recurrence will be 48%^6^. Therefore, the total sample was calculated to be 260 women with GDM, where 48% of them (125 women) had GDM recurrence. The sample size and power calculation was performed using SAS® %ROCPOWER macro^21^.

Categorical variables are presented using frequencies and percent. Continuous variables are presented using mean ± standard deviation [median]. The mean glucose values were calculated by dividing the sum of glucose measurements by the number of glucose measurements (for preprandial and postprandial glucose measurements the relevant measurements were considered in the calculation).

The ROC curve was used in order to evaluate the OGTT results and the mean glucose values variables accuracy (prevalence ratios (PRs) with 95% confidence interval (CI) were presented). The comparisons of the ROC curves were implemented using a nonparametric approach^22^. The optimal cut-off point to discriminate recurrence and non-recurrence GDM was chosen by calculating the ROC sensitivity and specificity pairs and choosing the pair with the minimal distance between them.

As odds ratios overestimate associations between risk factors and common outcomes (such as GDM recurrence) we used log-binomial regression instead of logistic regression^20,23^. Since the number of glucose reported days varied between the women, an adjustment was done by using multiple log-binomial regression. Multiple log-binomial regression was also used in order to estimate the adjusted PR of the mean glucose levels (adjustment was done for selected risk factors and possible confounders). When the model did not converge (a well-known problem of the log-binomial model), the COPY method was implemented^24^.

The non-parametric local regression (LOWESS smoothing) was used for presentation of the glucose levels throughout the gestational weeks. The statistical analyses were performed using SAS 9.4 software (SAS Institute Inc., Cary, NC, USA).

The study was approved by the Helsinki Ethics Committee of Emek Medical Center (approval number EMC0061-13) with informed consent waver. The datasets generated during and/or analyzed during the current study are available from the corresponding author on reasonable request.

## Results

A total of 426 women (Fig. 1) were included in the analysis, including 257 women (60%) that had GDM recurrence, and thus the statistical power increased to 95%. The characteristics of the 426 are presented in Table 1; the risk factors refer to the index pregnancy. IPI and BMI gain measure the change between the index pregnancy and the consecutive pregnancy. Aside from the 3-hour OGTT glucose levels (post 100 gr glucose load), HbA_1c_, neonatal birth weight, and the mean daily overall and preprandial glucose levels, all of the examined risk factors were significantly associated with GDM recurrence. The average number of daily glucose charts for each woman was 12 ± 10 with median of 9 charts (range 1–63 charts) and the average glucose measurements was 61 ± 55 with median of 42 (range 3–323). For each woman, the rate of glucose measurements per day was also calculated; the average number of measurements per day was 5 ± 1 with median 6 (range 1–7). Since each woman had a different number of daily glucose charts, we performed multivariable log-binomial regression and adjusted the mean glucose levels to the number of daily glucose charts. The mean postprandial glucose (adjusted for the number of daily glucose charts) was significantly associated with GDM recurrence (Table 1; PR = 1.89 [95% CI: 1.33, 2.73] for every 20 mg/dl increase).

### ROC curve for OGTT results and daily mean glucose charts

The mean daily glucose levels along with the OGTT levels were examined for accuracy in predicting GDM recurrence using the area under the ROC curve (AUC). The fasting and the 3-hour post 100 gr glucose load were not significant since their confidence intervals included AUC = 50% (54% [95% CI: 48%, 60%] and AUC = 49% [95% CI: 43%, 55%], respectively); hence they were not significantly different from random guessing. The 1- and 2-hour post 100 gr glucose load and the mean daily glucose were all significantly associated with GDM recurrence with relatively poor/fail accuracy (AUC = 59% [95% CI: 53%, 64%], AUC = 58% [95% CI: 52%, 63%], and AUC = 57% [95% CI: 51%, 63%], respectively). The results suggest that by maintaining a mean daily glucose of 94 mg/dl, the chance for GDM recurrence may decrease (with sensitivity 55% and specificity 57%). Moreover, women with 1-hour post 100 gr glucose load OGTT result higher than 195 are also at increased risk for GDM recurrence (with sensitivity 67% and specificity 46%). In addition, women with 2-hour post 100 gr glucose load OGTT result higher than 160 are also at increased risk for GDM recurrence (with sensitivity 56% and specificity 52%). Multiple log-binomial regression was implemented and the final model included: multiparity (*p* = 0.01; adj.PR = 1.24 [95% CI: 1.05, 1.47]), the 1-hour post 100 gr glucose load OGTT result (*p* < 0.0001; adj.PR = 1.05 [95% CI: 1.03, 1.07), insulin use (*p* < 0.0001; adj.PR = 1.39 [95% CI: 1.18, 1.62]), and IPI (*p* < 0.0001; adj.PR = 1.07 [95% CI: 1.04, 1.10]). The final model did not include the mean daily glucose (*p* = 0.64; adj.PR = 1.04 [95% CI: 0.88, 1.23]).

### Specified glucose levels during the index pregnancy

**Figure 2:**
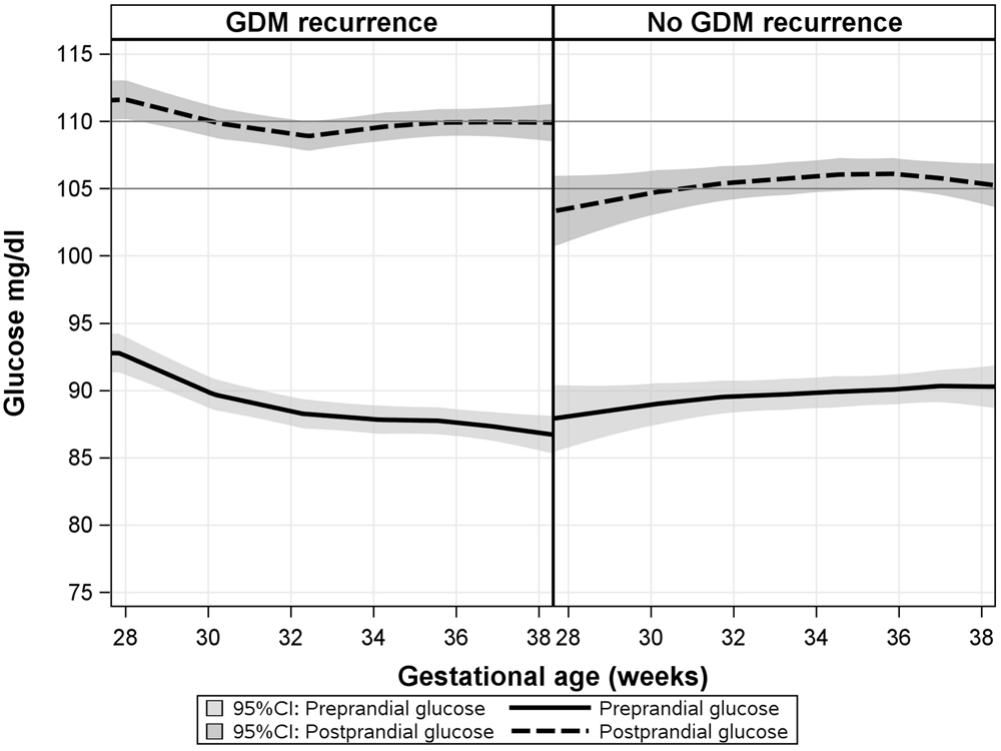
Association between gestational age and glucose levels among 303 women (4,226 daily glucose profiles), stratified by GDM recurrence, with LOWESS smooth for trend.

Of 426 women with mean glucose levels, 303 (71%) had specified glucose reports with information about the preprandial and postprandial glucose levels (Fig. 1). Approximately two thirds of the sample had glucose charts of a week or longer. The glucose levels trends along the gestational age (4,226 daily profiles), stratified by GDM recurrence status, are presented in Fig. 2. The figure trends showed no substantial differences between women with or without GDM recurrence regarding the preprandial glucose levels. Among women with GDM recurrence, the postprandial glucose levels were higher throughout the gestation weeks.

**Figure 3:**
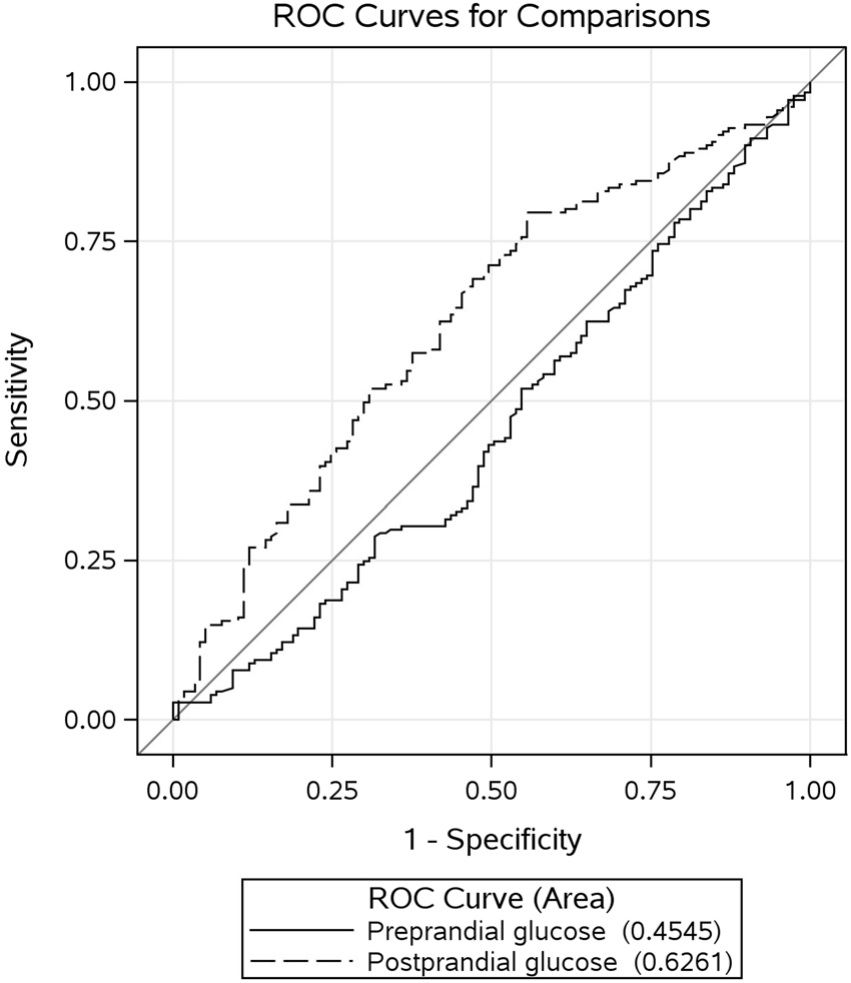
ROC curves for the mean preprandial and postprandial glucose levels in predicting GDM recurrence (N = 303).

The mean preprandial and postprandial glucose levels were also examined for accuracy in predicting GDM recurrence and the analyses results are presented in Fig. 3. Preprandial glucose was not significantly different from random guessing (AUC = 45% [95% CI: 39%, 52%]) and the postprandial glucose could predict GDM recurrence (AUC = 63% [95% CI: 56%, 69%]). The cutoff value of postprandial glucose reflecting GDM recurrence from the ROC analysis was 105 mg/dl for sensitivity and specificity of 64% and 56%, respectively. When a cutoff of 107 is considered, the sensitivity and specificity were 57% and 62%, respectively.

The mean preprandial glucose was not a predictor for GDM recurrence whereas the mean postprandial glucose was a significant predicator with 63% accuracy. Multiple log-binomial regression analysis was performed in order to obtain adjusted prevalence ratio of the mean postprandial glucose values. After adjusting for maternal age, multiparity, family history of diabetes mellitus, the fasting and 1-hour OGTT results, insulin use, IPI, BMI gain between the pregnancies, and the number of charts for each woman, the mean postprandial glucose levels remained significant (*p* = 0.017), with PR = 1.04 [95% CI: 1.01, 1.07; for every 20 mg/dl increase].

## Discussion

This study found that the postprandial blood glucose levels during pregnancy with GDM are significant predictors for GDM recurrence. Postprandial blood glucose is a known risk factor for neonatal hypoglycemia, macrosomia, and cesarean delivery^25^. Our study emphasized the role of postprandial glucose level as a risk factor for GDM recurrence. Unlike other risk factors, postprandial glucose level and glycemic control in general are the main markers for physicians when treating and managing GDM. Postprandial glucose could be reduced using intensive diet and hypoglycemic drugs treatment. For diet-controlled GDM women, it was found that a home-based cycling program helps to maintain the daily postprandial glucose^26^. Overall, by using strict postprandial glucose monitoring with lower target values, along with a recommendation to avoid long IPI and reducing weight, physicians may reduce the chance for GDM recurrence and, consequently, may reduce the chance for type 2 diabetes for some women.

Women with GDM have an increased risk for type 2 diabetes^27^. Kim *et al*^28^. found in their systematic review that the fasting glucose levels from OGTTs administered during pregnancy were predictive for type 2 diabetes, except in studies that also included more specific measures of β-cell function. The OGTT 1- and 2-hour results were also associated with future type 2 diabetes even when studies did examine β-cell function measures. Our results found that the OGTT 1- and 2-hour glucose levels (measured between 24 and 28 gestational weeks) were predictive of GDM recurrence with a consistent effect for the 1-hour result remaining in the multiple analysis final model. Although the mean daily glucose was predictive of GDM recurrence, the variable was not included in the final model.

This study has some limitations. A possible selection bias may have occurred due to unmonitored or missing glucose data. Women that were excluded due to unknown glucose values had significantly shorter IPI and lower GDM recurrence rate. As a result, they also gained less BMI between the pregnancies and were more likely to use diet as a means to control their glucose during the index pregnancy. These differences also may have caused an overestimation of the association between the glucose levels and GDM recurrence. However, in a systematic review that compared patient-generated blood glucose diary records with meter memory in diabetes^29^, it was found that among pregnant women, the mean meter blood glucose values were significantly higher than the diary values. This non-differential information bias is not associated with the GDM recurrence classification, and could have caused an underestimation of the association.

Although it had a significant effect, the mean postprandial glucose levels had poor accuracy in predicting GDM recurrence^30^ and the adjusted PR effect size was rather small. Nonetheless, we examined the postprandial glucose levels effect using three analysis methods and settings (mean postprandial glucose ROC and multiple log-binomial and glucose levels LOWESS trends), all of which found the postprandial glucose significant in predicting GDM recurrence. We concluded that there is a lasting effect on the level of postprandial glycemic control though its effect may not be strong.

## Conclusion

Our analyses included a large set of glucose charts during the third trimester of GDM pregnancies. To our knowledge, no study has examined the mean glucose level reports’ ability to predict GDM recurrence. The study conclusion is that postprandial levels during pregnancy have a lasting effect and can reflect the next pregnancy’s glycemic profile. Since GDM recurrence by itself is a risk factor for type 2 diabetes mellitus, it will be interesting to explore the postprandial levels during pregnancy as a risk factor for type 2 diabetes. In addition to the known modifiable risk factors^8^ (IPI and BMI gain between the pregnancies), this study now adds the postprandial glucose levels during the third trimester. By controlling the postprandial glucose levels (current guidelines ≤ 120 mg/dl), we may reduce the risk for GDM recurrence. Interventional studies are required to evaluate whether this is indeed a modifiable risk factor or it is a marker of a more severe insulin resistance. Either way, those women should be monitored carefully and strategies for GDM prevention should be implemented on this high risk population.
